# Orthodontic camouflage treatment for a patient with bilateral cleft lip and palate, bilateral crossbite, and microdontic maxillary lateral incisors

**DOI:** 10.1186/s40001-023-01589-3

**Published:** 2024-02-13

**Authors:** Lanxin Cheng, Kai Xia, Wentian Sun, Liyuan Yu, Zhihe Zhao, Jun Liu

**Affiliations:** Department of Orthodontics, State Key Laboratory of Oral Diseases & National Center for Stomatology & National Clinical Research Center for Oral Diseases, West China Hospital of Stomatology, Sichuan University, No. 14, 3Rd Section of South Renmin Rd, Chengdu, 610041 Sichuan China

**Keywords:** Cleft lip and palate, Malocclusion, Orthodontics, Mini-implant

## Abstract

Cleft lip and palate is a congenital craniofacial anomaly that affects the lip and oral cavity. The management and orthodontic treatment of this anomaly is important but challenging. This article reports the successful treatment of a patient with bilateral cleft lip and palate, Class III malocclusion, bilateral crossbite, crowding and microdontic maxillary lateral incisors. One mandible incisor was extracted, and three miniscrew anchorages were utilized to distalize the maxillary left dental arch and retract the mandibular arch. After treatment, ideal occlusion and a better profile were established, and long-term stability was confirmed by a 4-year follow-up. This article represents a successful attempt of orthodontic camouflage treatment of severe dentofacial discrepancy, as an important part of the series treatment of cleft lip and palate, to provide some insight into the clinical field.

Cleft lip and palate (CLP) is one of the most common congenital craniofacial anomalies. Males have a higher prevalence of CLP. The etiology of this anomaly is due to the incomplete development and fusion of the frontonasal prominence and maxillary and mandibular processes, caused by complicated molecular events resulting from genetic disorders and environmental risk factors, leading to severe functional and aesthetic problems [1]. Patients need to undergo complicated procedures from birth to adulthood, including cleft lip repair, cleft palate repair, alveolar bone grafting, orthognathic surgeries, etc. [2]

The management of CLP is multidisciplinary, among which orthodontics is an important but challenging component. CLP patients tend to have maxillary hypoplasia congenitally or caused by surgeries, and deformities of the skeleton often result in serious Class III malocclusions and crossbite. Protocols for maxillary expansion and protraction have exhibited positive outcomes for young-aged patients with growth potential. If perfect timing was missed, orthodontic camouflage treatment or surgery become the only options [3]. Orthognathic surgery would be an optimal option to correct skeletal disharmony, while camouflage treatment can only provide dentoalveolar compensation. Camouflage treatment involves the displacement of teeth to cover up jaw discrepancy, flaring of the maxillary incisors, retraction of the mandibular arch, lingual inclination of the mandibular incisors assisted by Class III elastics, extraction, mandibular arch distalization, etc. [4]

There are numerous barriers to the orthodontic treatment of CLP patients. Studies have shown that CLP patients exhibit abnormal buccolingual inclination of teeth that differs from the skeletal Class III malocclusion of non-CLP patients, which may be related to the multiple surgeries [5]. The undesirability restoration of alveolar bone defects makes orthodontic tooth movement very limited. Moreover, dental anomalies occur more frequently with CLP, among which dysgenesis of maxillary lateral incisors is the most common, and patients tend to have malformed or missing lateral incisors. The canines in addition to the alveolar cleft had statistically thinner bones than average [6]. A successful treatment for CLP patients requires comprehensive understanding and productive management of all the issues.

This case report represents a clinical case of orthodontic camouflage treatment for a CLP patient with Class III malocclusion, bilateral crossbite, and microdontic maxillary lateral incisors. After treatment, ideal occlusion and a better profile were established, and long-term stability was confirmed by a 4-year follow-up. This article represents a successful attempt of orthodontic camouflage treatment of severe dentofacial discrepancy, as an important part of the series treatment of cleft lip and palate, to provide some insight into the clinical field.

## Diagnosis and etiology

This 21-year-old male patient visited West China Hospital of Stomatology, Sichuan University, with a chief complaint of “underbite”. He had a history of bilateral complete cleft lip and palate and had received cheiloplasty at the age of 1, palatopharyngoplasty at 3 and secondary cleft lip repair at 19, but alveolar bone graft had not been performed. This patient claimed no family history and denied medical complications. The habit of tongue thrusting was identified.

**Fig. 1:**
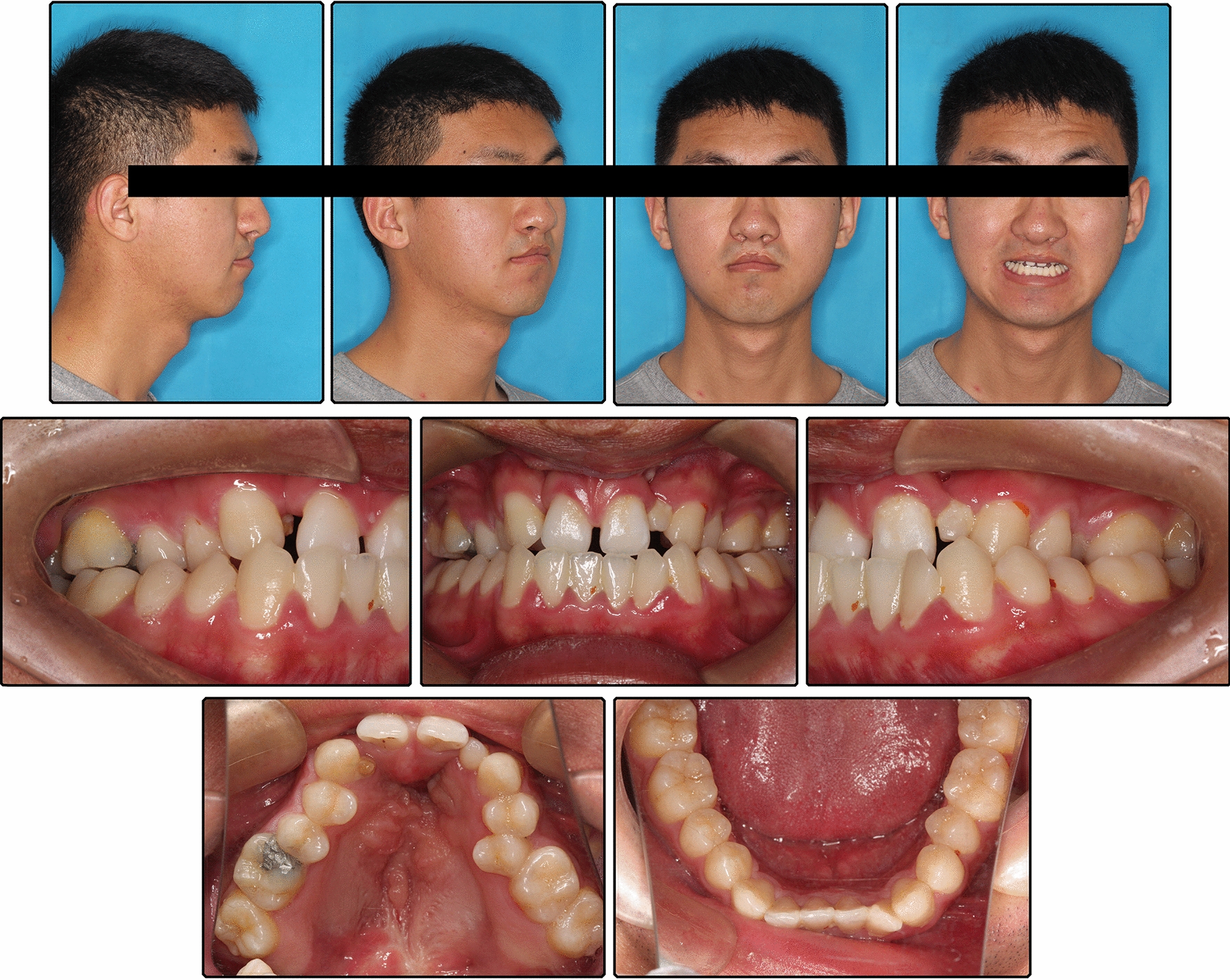
Pretreatment facial and intraoral photographs

**Fig. 2:**
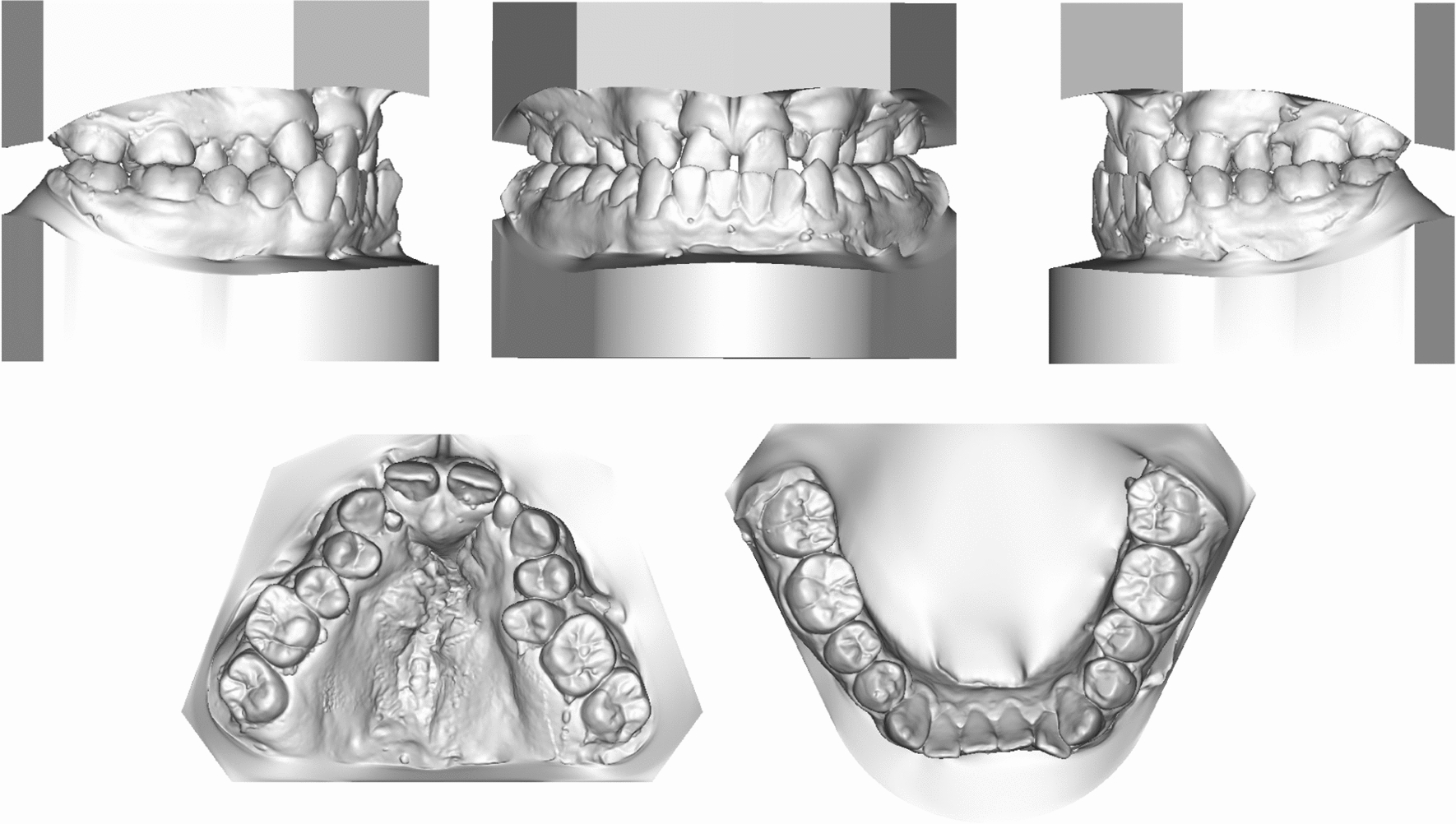
Pretreatment study models

Pretreatment facial photographs showed a concave profile with a protruded chin. The nasolabial angle was normal, and no facial asymmetry was noted. The mandibular dental midline was coincident with the facial midline, but the maxillary dental midline deviated to the right by 1 mm (Fig. 1). Intraorally, bilateral canines had a Class I relationship, the left molars had a Class III relationship, and the right molars had a Class II relationship, which may be attributed to the microdontia of the maxillary lateral incisors. Anterior and posterior crossbite was observed, but the mandible could be retruded to the edge-to-edge anterior tooth position. The anterior Bolton ratio was 89.60%, and the overall ratio was 97.16%. The maxillary lateral incisors were microdontic, and the maxillary right lateral incisor was extremely small with a conic shape, as many studies have demonstrated a high frequency of dental anomalies in patients with cleft lip and/or palate. [7] There was a small amount of space between maxillary anterior teeth, but severe crowding was observed in the maxillary dental arches with ectopic left second premolar (Fig. 2). Clinical examination of the temporomandibular joint showed no abnormalities.

**Fig. 3:**
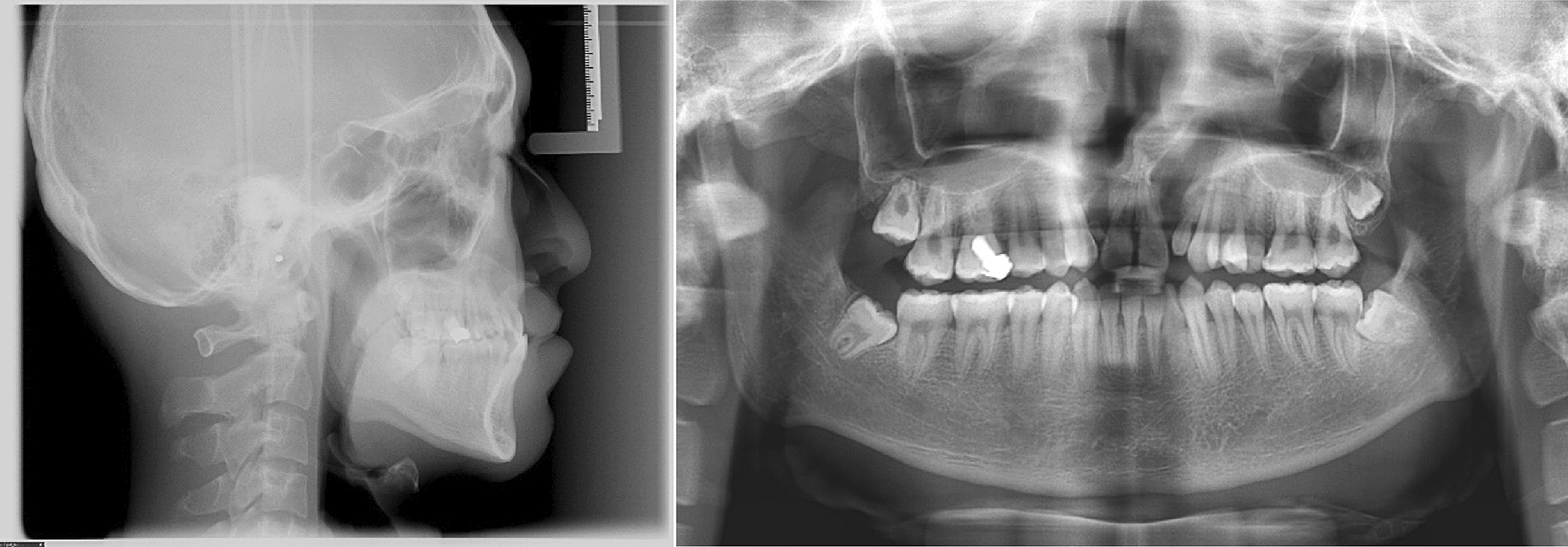
Pretreatment lateral radiograph and panoramic radiograph

**Fig. 4:**
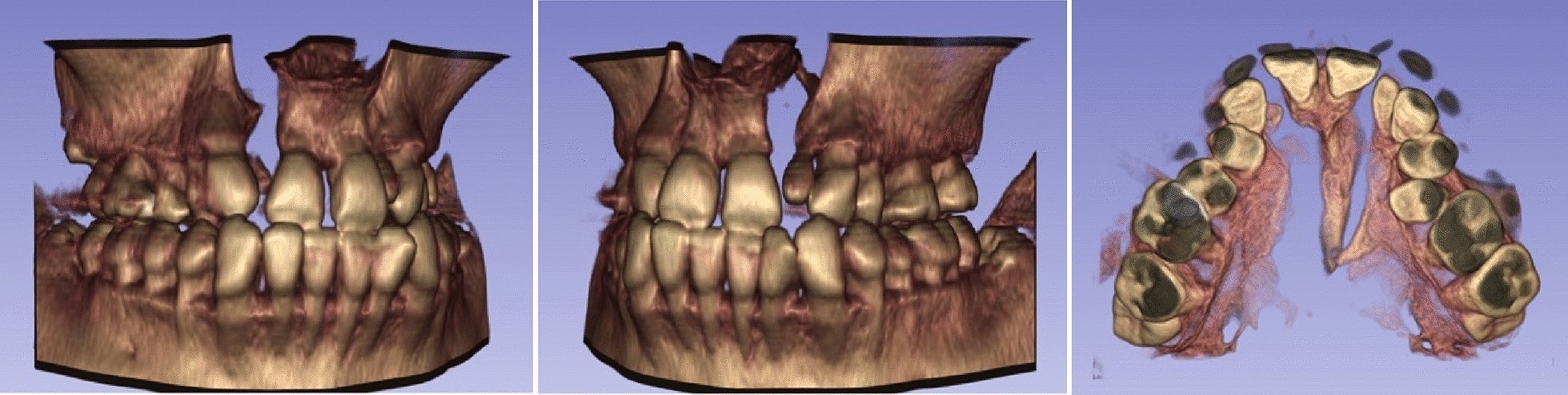
Pretreatment three-dimensional model reconstruction

Panoramic radiograph showed the residual root of maxillary right lateral incisor, fillings on maxillary right first molar, and impaction of third molars. Lateral cephalogram indicated a skeletal Class III malocclusion (ANB, 1.4°) (Table 1) and a hypodivergent vertical pattern (SN-MP, 29°) with large posterior to anterior facial height (S-Go/N-Me, 72.8%). The maxillary incisors were upright, and the mandibular incisors were lingually inclined (Fig. 3). The three-dimensional model reconstruction revealed a bilateral alveolar cleft (Fig. 4). This patient was diagnosed with post cheiloplasty and palatopharyngoplasty, bilateral alveolar cleft, skeletal Class III relationship, bilateral anterior and posterior crossbite, and severe maxillary crowding. According to the ABO discrepancy index, the complexity of this case measured up to 24 points (7 pts for anterior crossbite, 4 pts for crowding of 6 mm, 4 pts for Class III relationship on the right side and the Class II relationship on the left side, 5 pts for five posterior teeth in lingual crossbite, and 4 pts for two teeth with anomalous morphology) [8].

**Table 1:** Cephalometric measurements

Measurement	Norm ± SD	Pretreatment	Posttreatment
SNA (°)	82.7 ± 2.9	81.9	82.0
SNB (°)	79.7 ± 2.7	80.5	79.2
ANB (°)	3.0 ± 1.3	1.4	2.8
SN-MP (°)	34.9 ± 4.1	29.0	31.5
S-Go/N-Me	67.0 ± 4.0	72.8	70.1
U1-L1 (°)	124.9 ± 7.1	137.7	138.4
U1-SN (°)	107.2 ± 7.9	102.4	105.9
Ul-NA (°)	23.3 ± 6.2	19.1	22.1
UL-EP (mm)	− 0.1 ± 2.0	− 5.8	− 3.0
LL-EP (mm)	1.6 ± 2.2	1.2	0.6
Z angle (°)	71.8 ± 5.2	69.8	67.2

## Treatment objectives

The following treatment objectives were proposed: (1) align and level dental arches; (2) establish a dental Class I relationship; (3) establish normal overjet and overbite; (4) relieve crowding; (5) coordinate dental midlines; and (6) improve facial harmony and esthetics.

## Treatment alternatives

The best option for skeletal malocclusion would be combined surgical and orthodontic treatment. Indications for surgery include severe skeletal deformities: severe skeletal Class II or III malocclusion, mandibular deviation, facial asymmetry, et al. The patient exhibited severe arch width discrepancy and Class III malocclusion, nonsurgical treatment cannot address skeletal discrepancy, and the effect of compensation is limited. However, the patient rejected the surgical option out of fear of side effects, and orthodontic camouflage treatment was preferred for the patient. The severely deformed maxillary right lateral incisor was inevitably extracted, because its root was extremely short, as shown by the panoramic radiograph. There were two more nonsurgical options proposed.

The second option was to extract one mandibular incisor and all the third molars and create space to restore the maxillary right lateral incisor by implant or porcelain bridge. The Bolton ratio was higher than average, which indicates that for mandibular tooth-size excess, extraction of a mandibular incisor can be considered, and mandibular left lateral incisor was chosen to be extracted because it rotated greatly. One orthodontic mini-implant (OMI) was necessary on the left maxillary arch for molar distalization to create space for the ectopic premolar. Two more OMIs could be employed to retract the mandibular arch to correct Class III malocclusion.

The third option differed in the treatment of the microdontic lateral incisors: closing the space with a fixed appliance and reshaping the right canine to substitute the lateral incisor. This option would save the expense of prosthodontic treatment and alleviate the Bolton discrepancy. Class II malocclusion of the right side would reach a complete Class II relationship, whereas it can be regarded as a more stable occlusion. Nevertheless, this option would have a higher requirement on the control of anterior anchorage, while the alveolar cleft made it nearly impossible to implant OMIs in the anterior zone. Additionally, space closure would be dangerous for the teeth in addition to the cleft. Therefore, the second option was chosen.

## Treatment progress

The patient was suggested to visit an endodontist to evaluate if maxillary right first molar needed further treatment, as it had large fillings of amalgam. However, he refused the suggestion of root canal therapy and resin filling of it. The poor prognosis of the tooth was explained to the patient. Periodontal treatment and oral hygiene instruction were advised, and the habit of tongue thrusting needed to be eliminated.

**Fig. 5:**
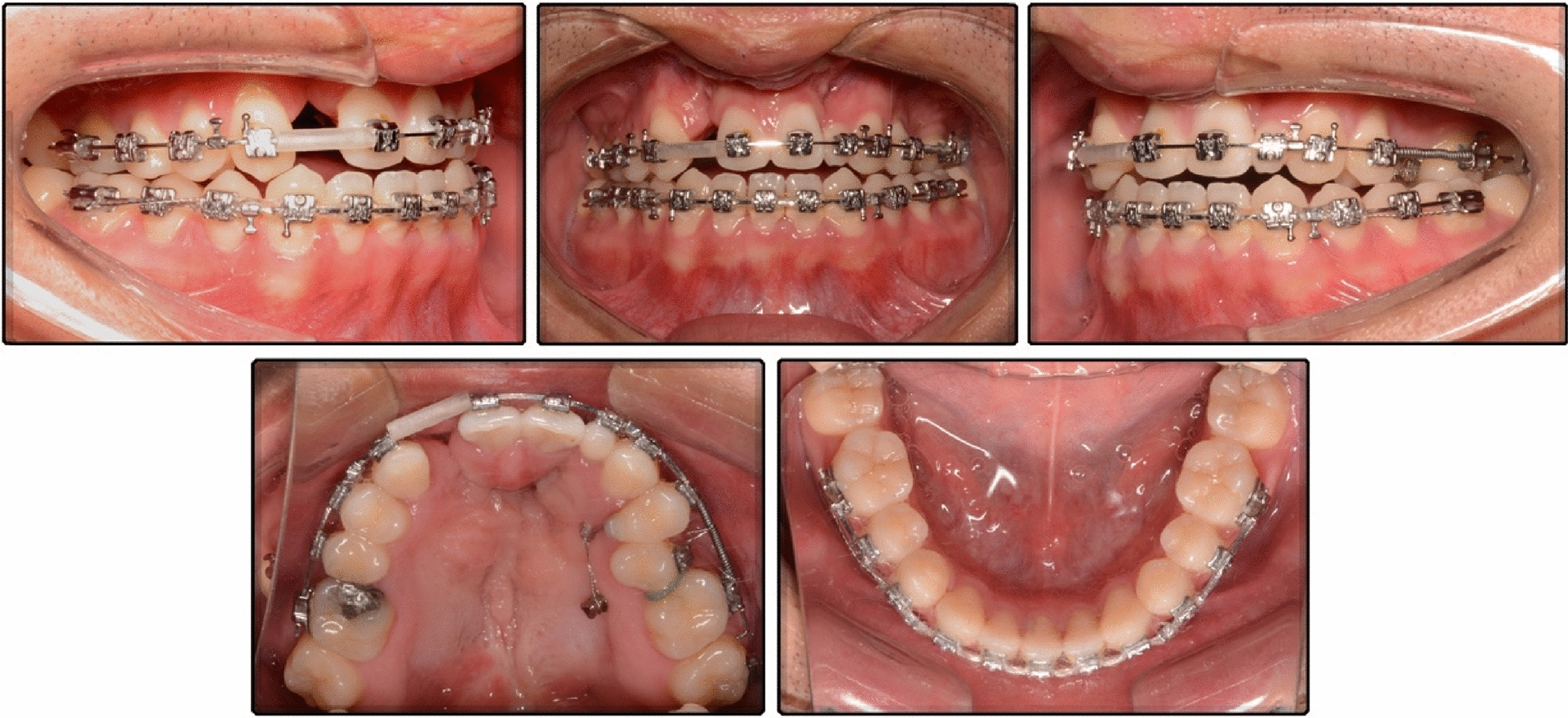
Initial alignment and leveling

Preadjusted MBT brackets, slot 0.022 -in (Masel, Ortho Organizers, Inc, 1822 Aston Avenue, Carlsbad, CA 92008) were used. A relatively high amount of torque in the maxillary anterior teeth would promote the labial inclination of the incisors. First, the brackets were cemented to the maxillary arch, and a biteplate was applied to prevent anterior occlusal interference. The mandibular arch was not cemented until the anterior crossbite was corrected. Nickel–titanium archwires (0.012, 0.014, 0.016, 0.018, 0.016*0.025 and 0.018*0.025) were sequentially applied to the dental arches to align and level the teeth. After 13 months of treatment, a 0.018*0.025-in stainless steel archwire was placed, a Ni–Ti coil spring was used to move the UR1 mesially to correct the maxillary dental midline while making space for maxillary right lateral incisor, and a plastic tube was placed on the archwire for space retention after enough space was gained. One OMI (1.4 × 8 mm, VectorTAS; Ormco Co, Brea, Calif) was implanted on the palate, sagittally between the root of maxillary left second premolar and first molar, and attached to the premolar through a long traction hook. A Ni–Ti coil spring was placed to create space for the second premolar. Maxillary left second premolar was ligated to the archwire through a traditional bracket bonded on the labial face, and a separator was placed between maxillary left second premolar and first molar. The OMI provided anchorage to distalize the maxillary left molars and prevented the anterior teeth from moving into the alveolar cleft (Fig. 5). It took 21 months to gain enough space for maxillary left second premolar and align it into the dental arch.

**Fig. 6:**
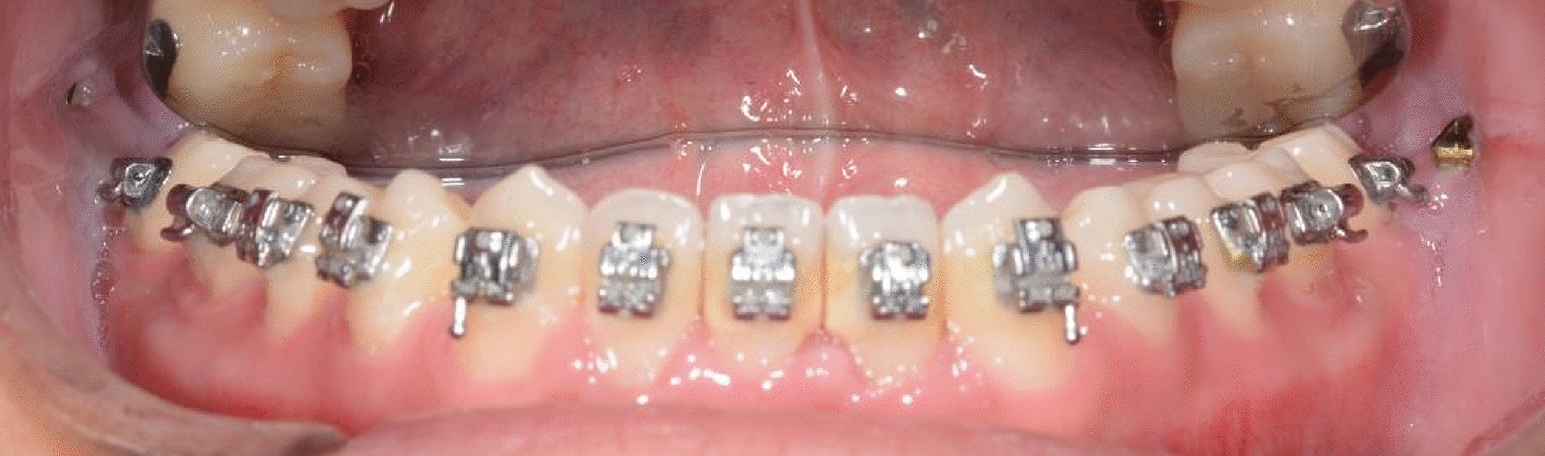
Implantation of mandibular OMIs

**Fig. 7:**
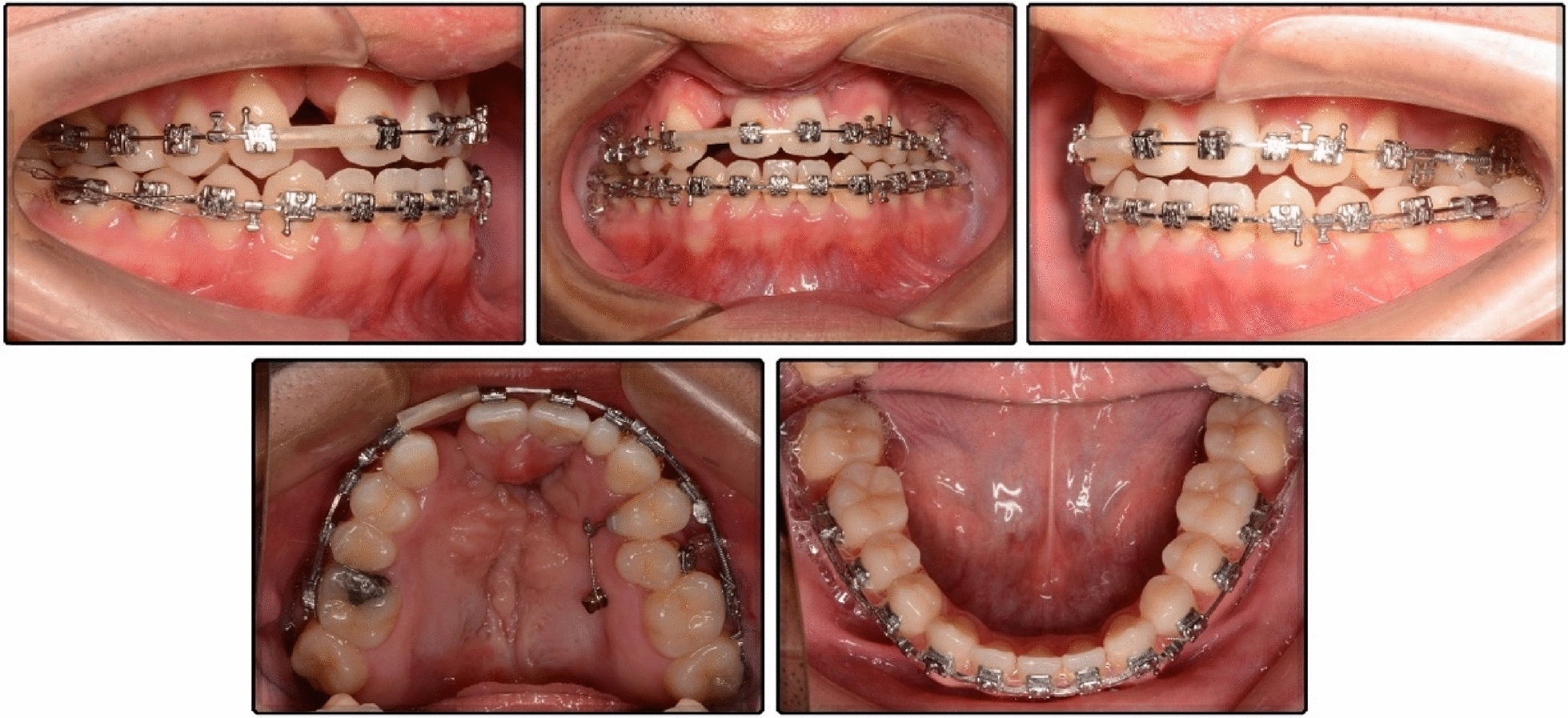
Mandibular retraction by OMIs

Mandibular space closure was achieved by elastic chains attached from mandibular right first premolar and left first premolar to retract the anterior teeth. We identified the dental caries of maxillary left first molar, and it was properly treated by the endodontist. Class III elastics (1/4 inch, 3.5 oz, Ormco Co, Brea, Calif) was utilized. On the 15th month, the space of mandibular left lateral incisor was completely closed, but the overjet was still not satisfactory. Therefore, two OMIs (2*12 mm, VectorTAS; Ormco Co, Brea, Calif) were inserted on the bilateral external oblique line for further retraction of the mandibular arch (Fig. 6). Elastic chains were attached from the OMI to the buccal tube of the mandibular second molars, and the mandibular arch was ligated as a whole (Fig. 7). After 30 months, elastic (3/16 inch, 3.5 oz, Ormco Co, Brea, Calif) was used for vertical traction in the anterior teeth to settle the occlusion.

In the 33rd month, appliances were removed, and the patient received a removable plastic retainer. The patient was informed that the retainer should be worn for at least three years, for whole day and whole night in the first year except dining and oral hygiene time, and the following two years only for night hours. Inappropriate retention may lead to relapse. A week later, the patient went to the Department of Prosthodontics and restored maxillary left lateral incisor by zirconia all-ceramic bridge.

## Treatment results

**Fig. 8:**
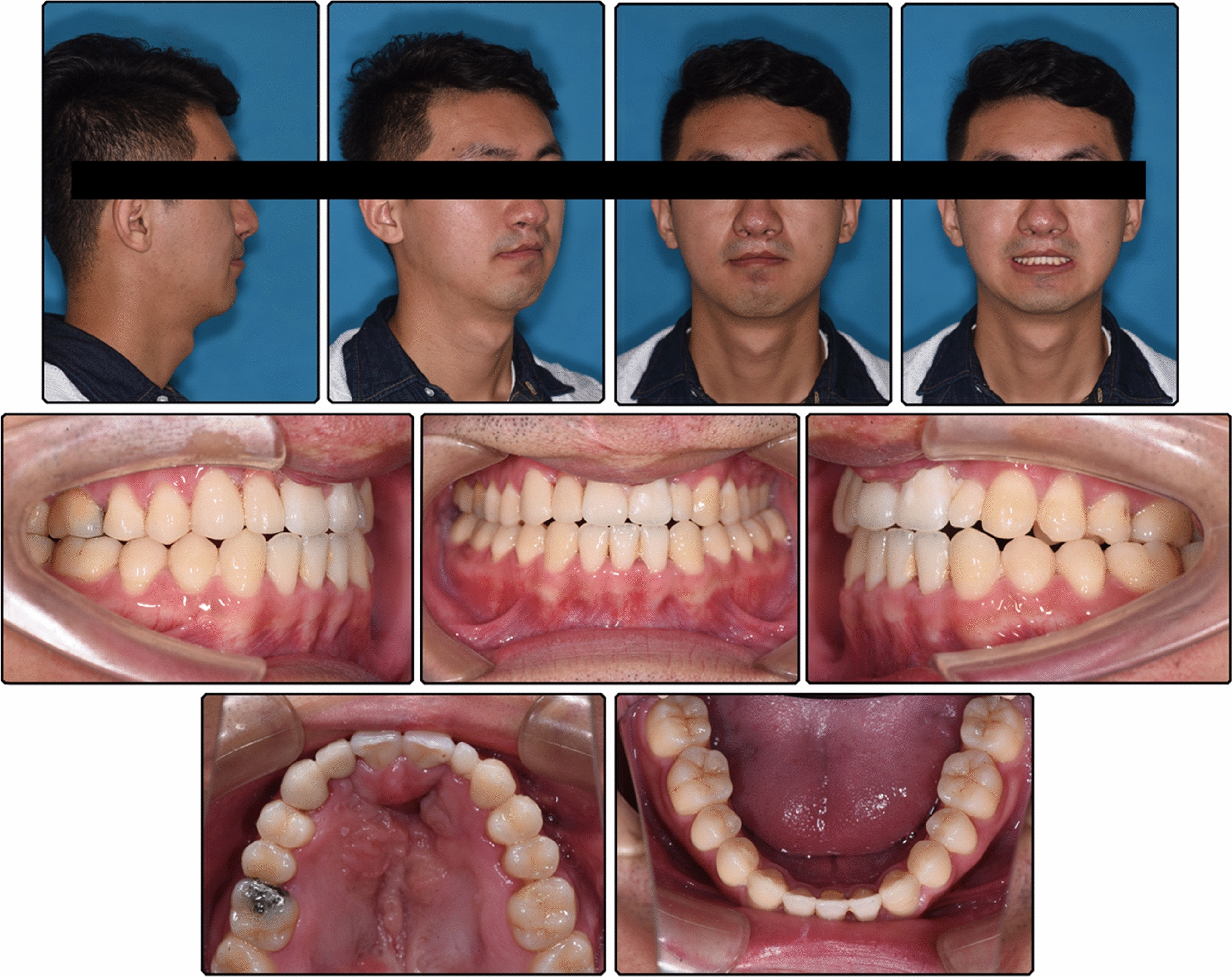
Posttreatment facial and intraoral photographs

**Fig. 9:**
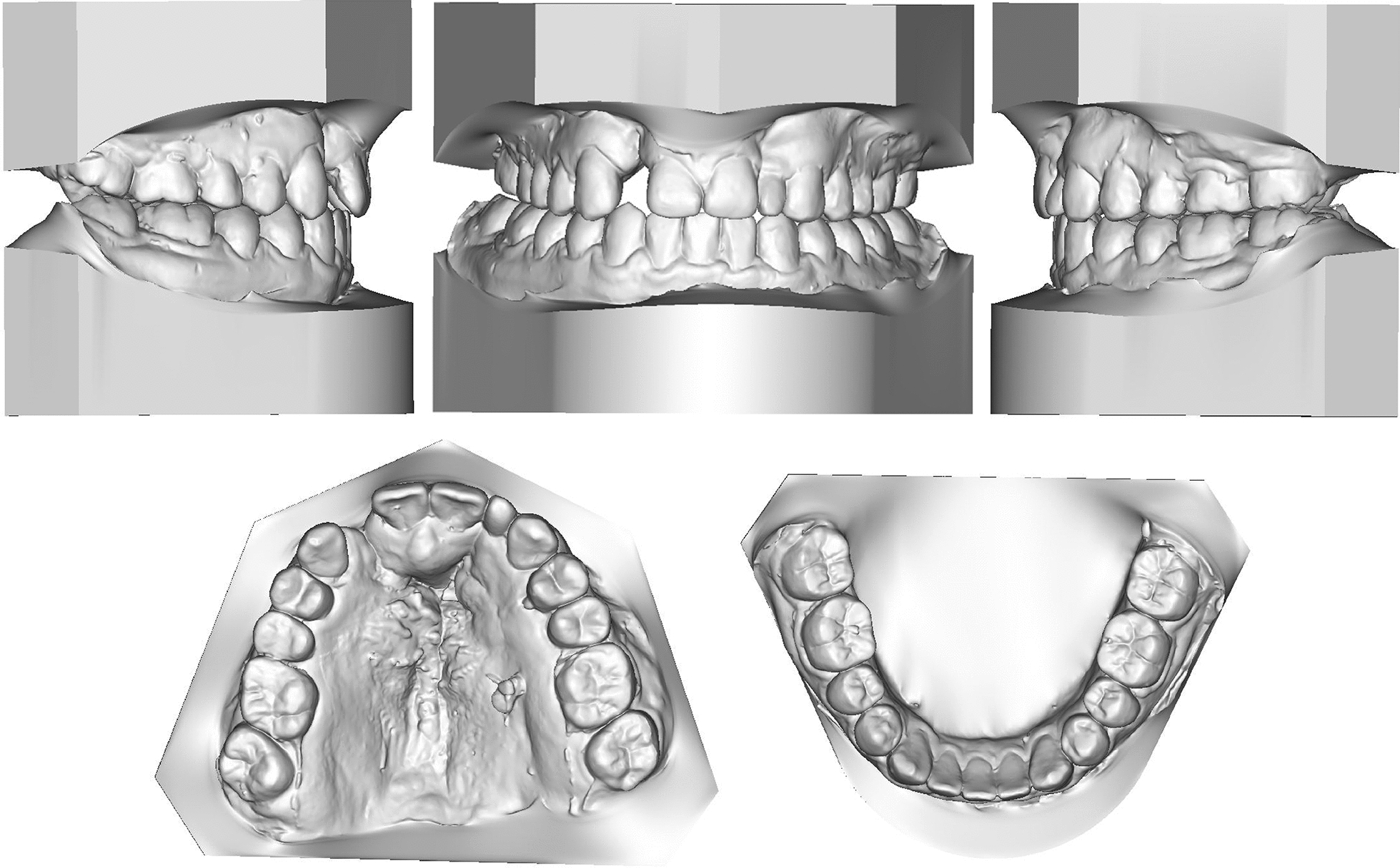
Posttreatment study models

After 33 months of treatment, right molars achieved a Class I relationship, and the left molars had a slightly Class III relationship, which was quite ideal considering the discrepancy of the jaw bones. The crossbite was corrected, the posttreatment overjet was 3 mm, and the overbite was 1 mm. Both arches showed good forms with well-positioned teeth in the bone. The maxillary midline coincided with the facial midline, and the midline of the mandibular arch turned to the midline of mandibular right incisor, which also coincided with the facial midline (Figs. 8, 9). Facial harmony and fine occlusion were achieved, and the patient was very satisfied with the outcome.

**Fig. 10:**
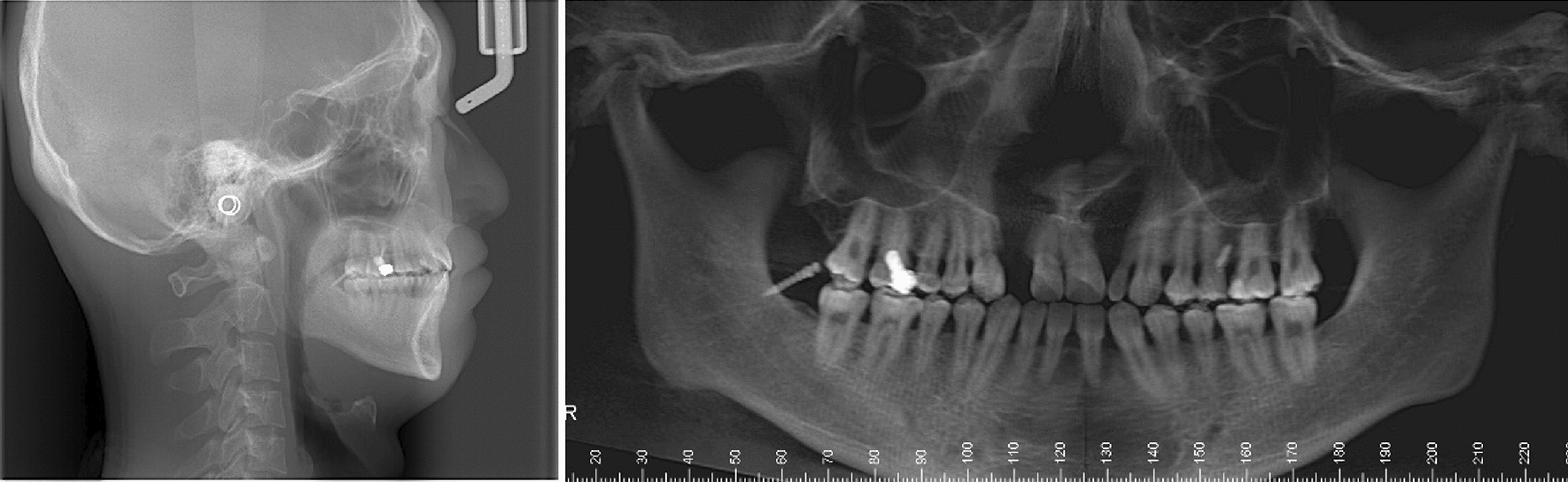
Posttreatment lateral cephalogram and panoramic radiograph

**Fig. 11:**
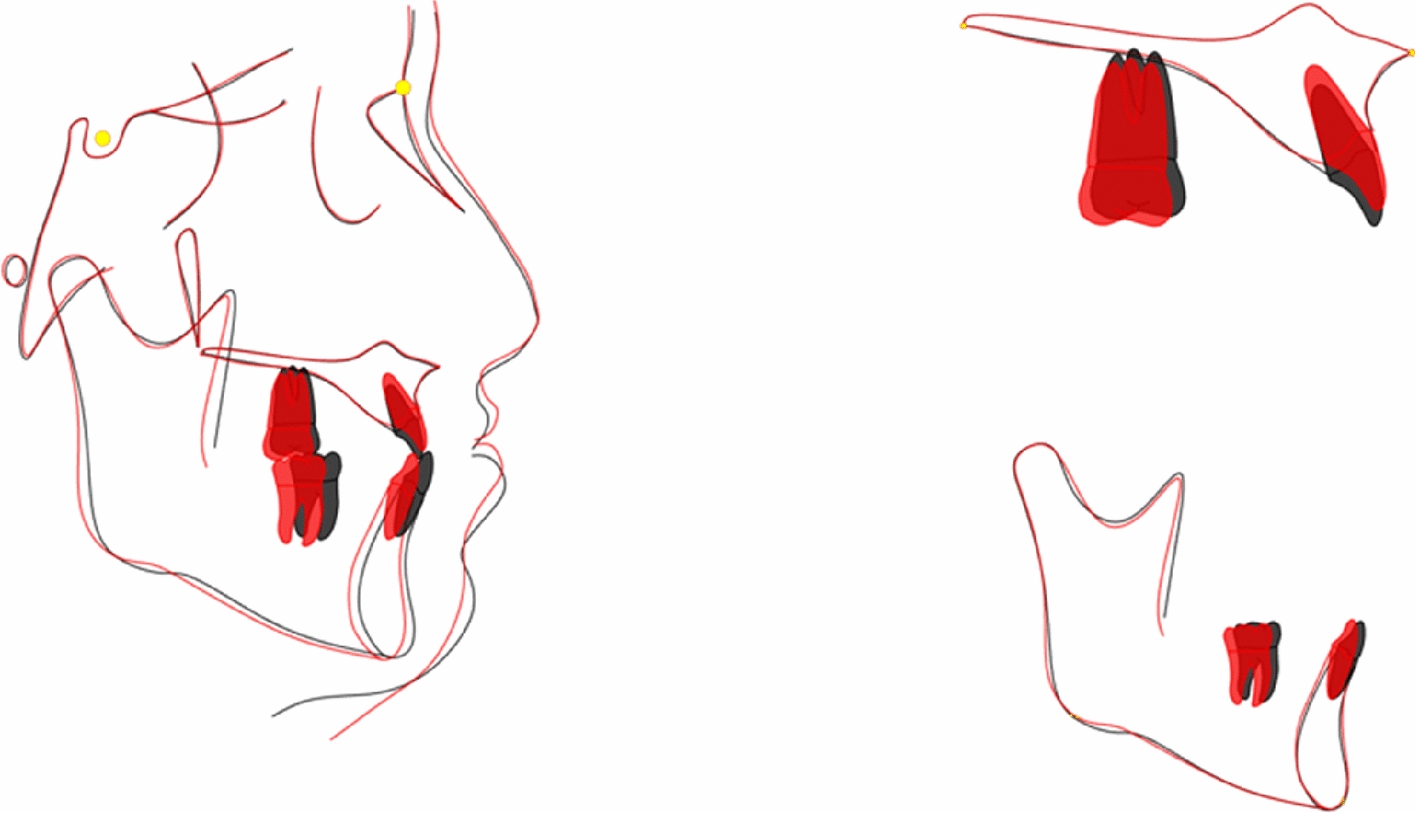
Superimposed tracings

**Fig. 12:**
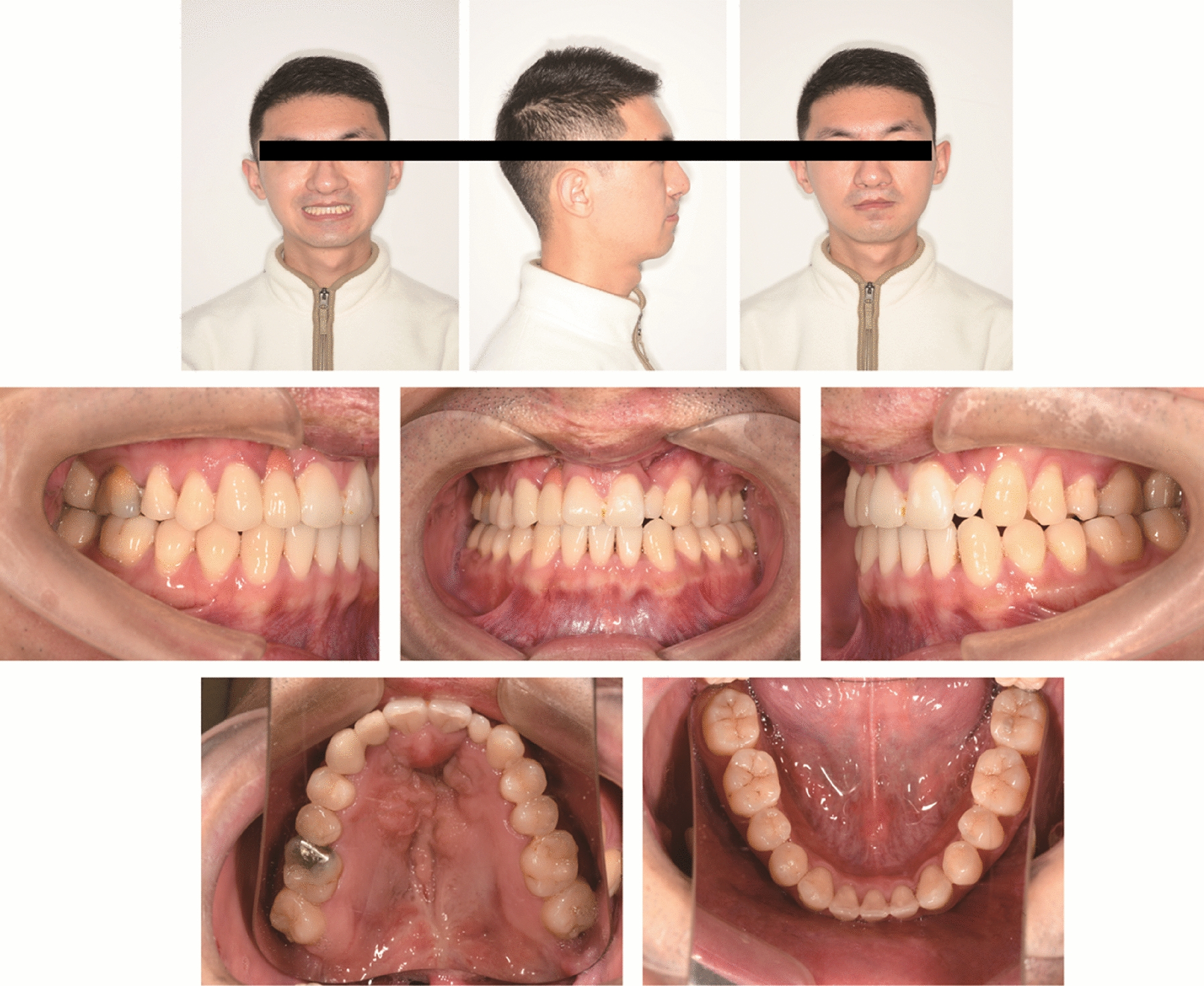
Four-year follow-up facial and intraoral photographs

Panoramic radiograph showed a parallel root, with no evident bone resorption. Lateral cephalometric tracing superimpositions showed clockwise rotation of the mandible, labial inclination of maxillary incisors, lingual inclination and intrusion of mandibular incisors and distalization of molars (Fig. 10, 11). The ANB angle increased from 1.4° to 2.8°, possibly because of the clockwise rotation of the mandible, which also greatly improved the profile. A better proportion of posterior and anterior facial height was achieved (S-Go/N-Me, 70.1). The soft tissue was positioned normally compared to the esthetic plane (UL-EP, -3.0 mm; LL-EP, 0.6 mm), which may be attributed to changes in the inclination of the anterior teeth. (Table 1). Furthermore, a 4-year follow-up shows the stability of the treatment (Fig. 12).

## Discussion

This case was successfully treated without surgery. A 6-mm arch width discrepancy was observed before treatment, and the maxillary arch was extremely narrow. It is worth mentioning that a narrow maxillary arch and crowding were proven to be associated with bad oral habits such as tongue thrusting, as the disequilibrium of muscle and hard tissues is highly likely to lead to malocclusions [9, 10]. The patient quits the habit of oral thrusting as advised, which had a positive effect on the treatment outcome. The dislocation of the tongue eliminated pressure on the mandibular arch while providing stimulation to the maxillary arch. Chewing muscles, dentition and jaw bones are closely connected as a whole, having significant impact on each other. To master the relationship between soft and hard tissue can yield twice the result with half the effort during the treatment.

It is widely acknowledged that orthodontic treatment for CLP patients is very challenging for many reasons. A satisfying outcome requires solid orthodontic skills and a comprehensive understanding of the malocclusion of CLP patients. First, the skeletal malocclusion of CLP patients tends to be more serious because of the growth insufficiency of the maxilla with or without over growth of the mandible. Although orthodontic treatment has a limited effect on jawbones, especially for adults with no growth potential, dentoalveolar compensation can be of some help. Normative cephalometric data are important reference standards for orthodontic diagnosis, and it’s worth mentioning that cephalometric measurements of the face and cranial base differ between genders [11]. As the patient had a basically normal nasolabial angle, mandibular retraction should be the main approach to attain a better profile. Movements of mandibular anterior teeth should be monitored carefully, as studies have reported decreased bone thickness in the mandibular anterior region for Class III malocclusion patients [12]. 3D reconstructions of the craniomaxillofacial region using cone beam computed tomography (CBCT) can be employed for the morphological evaluation of specific anatomical structures, which are extremely useful in the diagnosis and planning of orthodontic treatment. Studies have confirmed the accuracy of different types of software for the semiautomatic segmentation of the mandibular jaw, thus suggesting that the 3D rendering of jaw bones was highly reliable to evaluate the craniofacial morphology [13]. In this case, CBCT showed thin buccal cortical bone for mandibular incisors, and further labial movement of the roots may lead to bone fenestration or dehiscence. Torque control was given considerable attention throughout the treatment. Brackets with a high amount of torque were utilized, and thick archwires promoted the expression of torque. A reverse curve of the Spee archwire was also employed for anterior labial inclination. Superimposed tracings revealed little root movement but notable lingual inclination of crowns for the mandibular incisors, which indicated good torque control. Anchorage control is another important factor. Two OMIs were employed, which provided absolute anchorage for whole-arch retraction, and mandibular molar distalization was successfully achieved. The wedging effect of the distalization may lead to clockwise rotation of the mandible, thereby alleviating Class III malocclusion and leading to improvements in occlusion and profile.

Second, CLP patients tend to have defective periodontal conditions; for instance, the existence of an alveolar cleft would bring barriers to orthodontic tooth movement. Alveolar bone graft is one part of the treatment protocols for CLP patients, yet there remain many uncertainties in the treatment protocol, such as the timing of repair, surgical designs and whether to use presurgical orthopedic techniques [14]. In addition, the costs of time, energy and money are enormous. Very few CLP patients have been well treated. Our patient failed to receive alveolar bone graft, and the cleft between maxillary right incisor and canine, left incisor and lateral incisor can be observed. The teeth in addition to the cleft must be monitored carefully in case they move into the cleft, which may compromise tooth vitality, and teeth too close to the cleft may result in gingival recession [15]. Therefore, we used a plastic tube on the archwire at the place of maxillary right lateral incisor, which can prohibit incisor and canine from moving toward the cleft. At the left side, an OMI was employed to keep the anterior teeth from moving to the cleft while creating space for maxillary right second premolar. Care was taken for the torque of maxillary incisors as the roots were near the cleft, and too much palatal movement of the roots may be dangerous.

Another factor that increases difficulty for the orthodontic treatment of CLP patients is the occurrence of dental anomalies. It was demonstrated that CLP patients have a higher incidence of dental anomalies, which may be attributed to the proximate anatomy and closely related development of teeth and palate [16]. Tooth agenesis, ectopic eruption and microdontia are more frequently observed, and anomalies of the lateral incisor have the highest prevalence [17]. Microdontic teeth can be managed in many ways, depending on the severity of microdontia and the wishes and requirements of the patients. Prosthodontic treatment is the most common method, and orthodontic treatment is usually needed to create enough space for restoration. Nevertheless, it is undesirable to blindly restore the tooth to its normal form and function, and many factors should be taken into account. In this case, it was considered that the microdontic maxillary left lateral incisor had a short root, and it was near the cleft. If restored to its normal size, the increase in occlusal force may lead to its loose or root fracture. Therefore, maxillary left lateral incisor did not undergo further restoration.

After four years of follow-up, there was no relapse of crowding, and overjet and overbite remained normal. Long-term stability was confirmed, for which good compliance with retainer wearing is crucial. Retention is also a crucial stage of orthodontic treatment, the outcome of which depends on the type of malocclusion, treatment protocol, retention plan and compliance of the patient [18]. Stability can be particularly poor when arch expansion is conducted, as the equilibrium of oral soft and hard tissues is disrupted, which may exert pressure on the teeth until periodontium remodeling is completed. Therefore, retainers are crucial to hold the teeth in their position [19]. Many other factors through the treatment can also be taken into account to enhance stability; one is to place teeth roots upright in basal bone, and the decompensation that uprighted the severely labially or lingually inclined teeth can be of great help to improve stability. The arch form and intercanine width are also important for stability, and overexpansion should be avoided. In our case, the pretreatment arch form was incompatible with the basal bone; hence, it was appropriate to expand the arch form accordingly. Ideal occlusion and function are also keys for posttreatment stability, and bad oral habits should be corrected [20].

## Conclusions

We reported the successful treatment of a CLP patient with skeletal Class III malocclusion, bilateral crossbite, crowding and microdontic maxillary lateral incisors. With the help of digital software, craniofacial morphology was assessed and treatment plan was set. Single mandibular extraction, arch expansion and mandibular retraction were applied, and OMIs were of great help in the treatment. Ideal occlusion and an improved profile were attained, and long-term stability was affirmed. Orthodontic treatment for CLP patients is unique and difficult, and with appropriate treatment and retention protocols, successful outcomes and long-term stability are possible.

## Data Availability

Not applicable.
